# Capacity differences in working memory based on resting state brain networks

**DOI:** 10.1038/s41598-021-98848-2

**Published:** 2021-09-30

**Authors:** Mariko Osaka, Mizuki Kaneda, Miyuki Azuma, Ken Yaoi, Tetsuya Shimokawa, Naoyuki Osaka

**Affiliations:** 1 Center for Information and Neural Networks, Advanced ICT Research Institute, National Institute of Information and Communications Technology, 1-4 Yamadaoka, Suita City, Osaka 565-0871 Japan; 2 Research Center for Child Mental Development, Kanazawa University, 13-1 Takaramachi, Kanazawa-shi, Ishikawa 920-8640 Japan; 3 Department of Psychology, Graduate School of Letters, Kyoto University, Yoshida-honmachi, Sakyo-ku, Kyoto 606-8501 Japan

**Keywords:** Working memory, Cognitive control

## Abstract

Herein, we compared the connectivity of resting-state networks between participants with high and low working memory capacity groups. Brain network connectivity was assessed under both resting and working memory task conditions. Task scans comprised dual-task (reading sentences while memorizing target words) and single-task (reading sentences) conditions. The low capacity group showed relatively stronger connectivity during resting-state in most brain regions, and the high capacity group showed a stronger connectivity between the medial prefrontal and posterior parietal cortices. During task performance, the dorsal attention and salience networks were relatively strongly connected in the high capacity group. In the comparison between dual- and single-task conditions, increased coupling between the anterior cingulate cortex and other attentional control-related areas were noted in the high capacity group. These findings suggest that working memory differences are related with network connectivity variations in attentional control-associated regions during both resting and task performance conditions.

## Introduction

Working memory involves the temporary storage and processing of information, and supports higher cognitive functions^1–3^. Information held in working memory remains in an active state and can be processed until the task goal is reached. The working memory capacity has constraints, and when a task pushes its resources toward their limits, individual capacity differences can be observed. For example, individual differences have been observed during tasks requiring dual processes to achieve task goals^4–7^. Furthermore, individual differences in working memory have various effects on higher cognitive functions^2,8^. Additionally, working memory capacity has been suggested to reflect the central executive capability because it serves as an attention controller, allocating and coordinating attentional resources for cognitive tasks^9,10^.

The neural basis of central executive functions depend on regions such as the dorsolateral prefrontal cortex (DLPFC) and anterior cingulate cortex (ACC)^11–16^. We previously investigated the neural basis of individual differences in the performance of complex span tasks, such as the reading span test (RST) and listening span test (LST), which measure capacity differences in the memorizing of target words while reading (RST) or listening (LST) to a few sentences. DLPFC and ACC activity was strongly enhanced in both conditions. We also identified individual differences in DLPFC and ACC activation: participants with high capacity had a stronger magnitude of increase in activity in both these regions than participants with low capacity^14,15^. Moreover, these two regions were more strongly and positively activated during the complex span tasks in the high capacity group than in the low capacity group^14,15,17^.

We also compared two types of RSTs: focus and non-focus. In the former, the target word to be memorised was an important word for sentence comprehension, whereas in the latter, it was not; thus, attention shifting was required. Compared to in-focus RST, an enhancement in the superior parietal lobule (SPL) was observed in the non-focus RST. Moreover, a between-group difference in SPL enhancement was noted, with a greater increase in activation in the high capacity group than in the low capacity group^18,19^. Based on these results, we conjectured that the neural substrates underlying individual differences in executive function depend on the cooperation of the DLPFC, ACC, and SPL^18^.

An unanswered question is how individual differences in working memory capacity are underpinned by the dynamics of large-scale neural networks. Recent neuroimaging studies suggest that the human brain is organised into distinct functional networks that support cognitive processes^20–22^. Based on resting-state functional connectivity studies*,* the existence of four main large-scale networks has been suggested: the default-mode network (DMN), dorsal attention network (DAN), frontal parietal network (FPN), and salience network (SN). The DMN is strongly activated at rest, while the DAN and FPN are activated during the performance of attention-demanding cognitive tasks^23–26^, and the SN is involved in the detection and filtering of salient stimuli^21,27^. Moreover, network studies have suggested that components of the DMN are activated negatively with the DAN, and anti-correlations between these networks have been reported^28,29^, which may involve the SN switching between the two networks^30,31^.

Recent researches have also suggested a relationship between resting-state network connectivity and the executive function of working memory, as reduced medial prefrontal cortex (MPFC)-posterior cingulate cortex (PCC) connectivity within the DMN correlates with poor executive function performance^32,33^. In addition, the anti-correlation between the MPFC (in the DMN) and DLPFC (in the DAN) is positively correlated with working memory task performance during n-back tasks^34^, letter-number sequencing subtest (WAIS-III)^35^ and dual task performance^36^. It is also interesting that the inability to anticorrelate is involved in cognitive impairment^37,38^. Thus, an anti-correlation between the DMN and DAN and positive correlations within the DMN may underlie the working memory capacity.

However, the neural mechanisms underlying this anti-correlation between the DMN and DAN remain unclear. In addition, how resting-state networks differ among individuals with working memory capacities, not only under resting-state conditions, but also during the performance of dual tasks requiring executive functions, remains unsolved.

Thus, we aimed to identify the brain networks involved in the executive function of working memory by comparing brain network connectivity under resting and working memory task conditions, and between participants with high and low capacity using the complex span test (a measure of the executive function of working memory). Therefore, this study compared the network connectivity between a high capacity group (HCG) and a low capacity group (LCG) of working memory, while at rest and while performing a working memory task. Furthermore, to clarify the effect of executive function of working memory on connectivity differences, the two groups were scanned during a dual-task condition that required working memory executive function (RST condition), and a single-task condition that did not (READ condition). Considering the capacity differences of the executive control of working memory, the differences should be greater in dual-task condition as the dual task requires greater executive control than single task conditions^9,10,19^.

## Results

We compared the behavioural performance of HCG and LCG by two-sample t-test, using IBM SPSS Statistics 25. The alpha level was 0.05. We conducted Levene’s test for equality of variances, and if the result of Levene’s test was significant (*P* < 0.05), then we adopted the result of t-test in case of ‘equal variances not assumed’.

### Behavioural performance before functional magnetic resonance imaging (fMRI)

**Figure 1:**
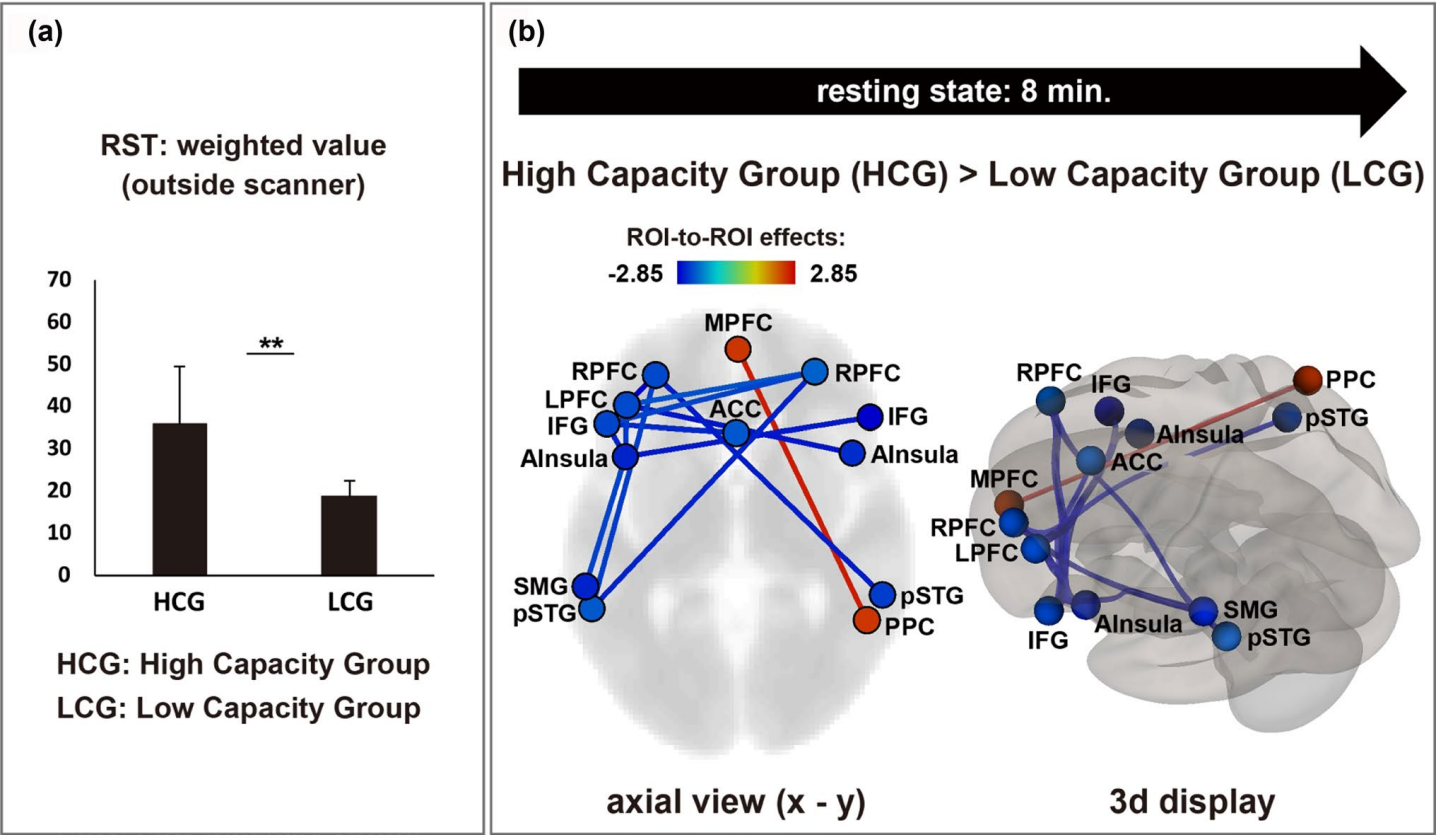
Working memory capacity and network connectivity during rest. (**a**) The weighted values of the reading span test of two groups. (**b**) Network connectivity during the resting-state condition. The left and right sides of the figure show an axial and three-dimensional display of the brain, respectively. Red lines indicate higher connectivity in the HCG than in the LCG ((uncorrected for multiple comparisons, *p* < .05). Blue lines indicate higher connectivity in the LCG than in the HCG ((uncorrected for multiple comparisons, *p* < .05).

Working memory capacity was estimated by three RST indices measured outside the scanner: weighted value^7^, total recall score, and span score^4,14^. Based on the RST results of weighted value and total recall score, we divided 34 participants into the following two groups with 17 participants each: HCG and LCG. For the weighted value, the mean score was 35.76 (23–66, *SD* = 13.64) in the HCG and 18.71 (11–23, *SD* = 3.72) in the LCG (*t* (18.4) = 4.98, *p* < 0.001), as shown in Fig. 1a. For total recall, the mean score was 56.18 (49–69, *SD* = 6.29) in the HCG and 43.24 (32–51, *SD* = 4.41) in the LCG (*t* (32) = 6.95, *p* < 0.001). For the span score, the mean score was 3.41 (2.5–5.0, *SD* = 0.92) in the HCG and 2.77 (2.0–3.0, *SD* = 0.36) in the LCG (*t* (20.7) = 2.70, *p* < 0.05). All indices indicated that the working memory capacity significantly differed between the two groups. In contrast, the two groups did not show a significant difference in the scores of the word span test (WST), a single task which only required remembering memorised words; the mean span score was 4.41 (3.5–7.0, *SD* = 0.82) in the HCG and 4.03 (3.0–4.5, *SD* = 0.48) in the LCG (*t* (32) = 1.66, *ns*).

### Behavioural performance during task fMRI

**Figure 2:**
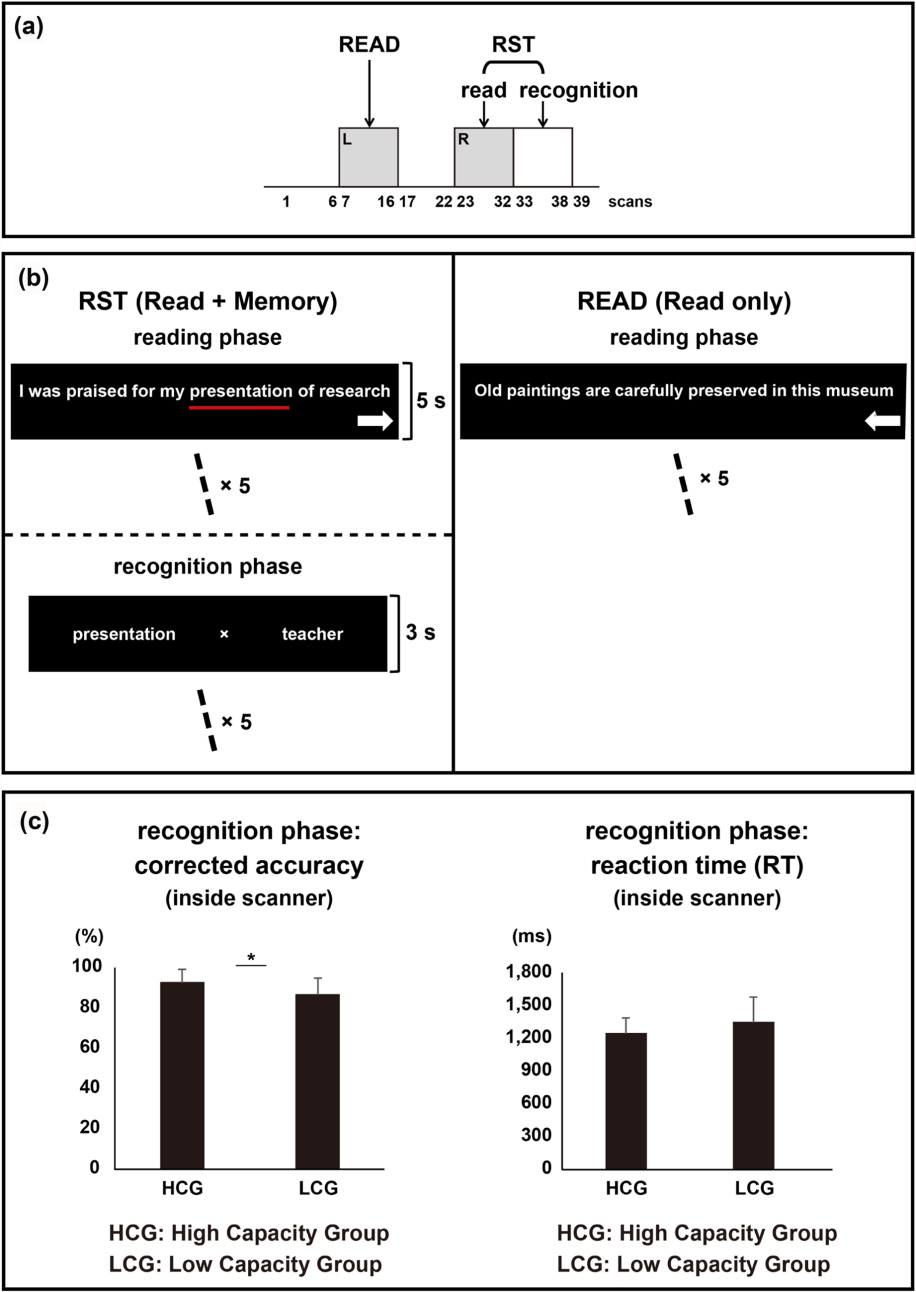
Experimental paradigms and behavioural results of RST condition. (**a**) Experimental time course of RST and READ condition. (**b**) The left side figure shows the RST experimental paradigm of reading and recognition phases. The right side of the figure shows the READ experimental paradigm which comprises only a reading phase. (**c**) Recognition accuracies (left side) and reaction times (right side) of RST condition in high capacity group (HCG) and low capacity group (LCG).

Figure 2a shows the time course of the RST and READ conditions. During the RST performed in the scanner, participants silently read five sentences and memorised target words underlined within the sentences during the reading phase, which was followed by the recognition phase, wherein participants were required to judge which word was the target word (see Fig. 2b; left side). For comparison with RST dual-task condition, participants were also scanned under a single-task condition (READ), in which they only read five sentences (see Fig. 2b; right side). The behavioural performance in terms of recognition accuracy and reaction time (RT) was measured (see Fig. 2c) during RST condition. A significant difference between groups was observed for recognition accuracy; the mean percent accuracy was 92.21 (*SD* = 6.84) in the HCG and 86.32 (*SD* = 8.48) in the LCG (*t* (32) = 2.23, *p* < 0.05). The RT did not significantly differ between the two groups; the mean RT was 1242.11 ms (*SD* = 147.59) in the HCG and 1347.64 ms (*SD* = 230.17) in the LCG (*t* (32) = 1.59, *ns*).

Under both conditions, participants were instructed to press a key corresponding to the arrow direction (shown at the bottom of right side of each sentence; Fig. 2b) after reading each sentence. The time required to read the sentences was measured, however, they did not significantly differ between the groups. Under RST condition, the mean RT was 3034.72 ms (*SD* = 713.02) in the HCG and 2903.58 ms (*SD* = 599.11) in the LCG (*t* (32) = 0.58, *ns*). Under READ condition, the mean RT was 2778.12 ms (*SD* = 685.10) in the HCG and 2809.27 ms (*SD* = 578.96) in the LCG (*t* (32) = 0.14, *ns*).

### Behavioural performance after the fMRI experiment

To further ensure that the participants read the whole sentences, a recognition test of the stimulus sentences was performed after the fMRI scan. The mean recognition accuracy was 25.18 (*SD* = 3.91) in the HCG and 24.24 (*SD* = 2.49) in the LCG (*t* (32) = 0.84, *ns*), not significantly different.

### Connectivity analysis

Figure 1b shows the network connectivity under the resting-state condition, during which the participants rested for eight minutes with their eyes closed. Significant group differences were observed. The network connectivity among most of the regions was stronger in the LCG than in the HCG (uncorrected for multiple comparisons, *p* < 0.05). However, the MPFC (in the DMN) and right posterior parietal cortex (PPC, in the FPN) were more strongly connected in the HCG than in the LCG (uncorrected, *p* < 0.05). In the present study, in order to clarify the details of individual differences, we selected comparisons applying uncorrected criterion.

**Figure 3:**
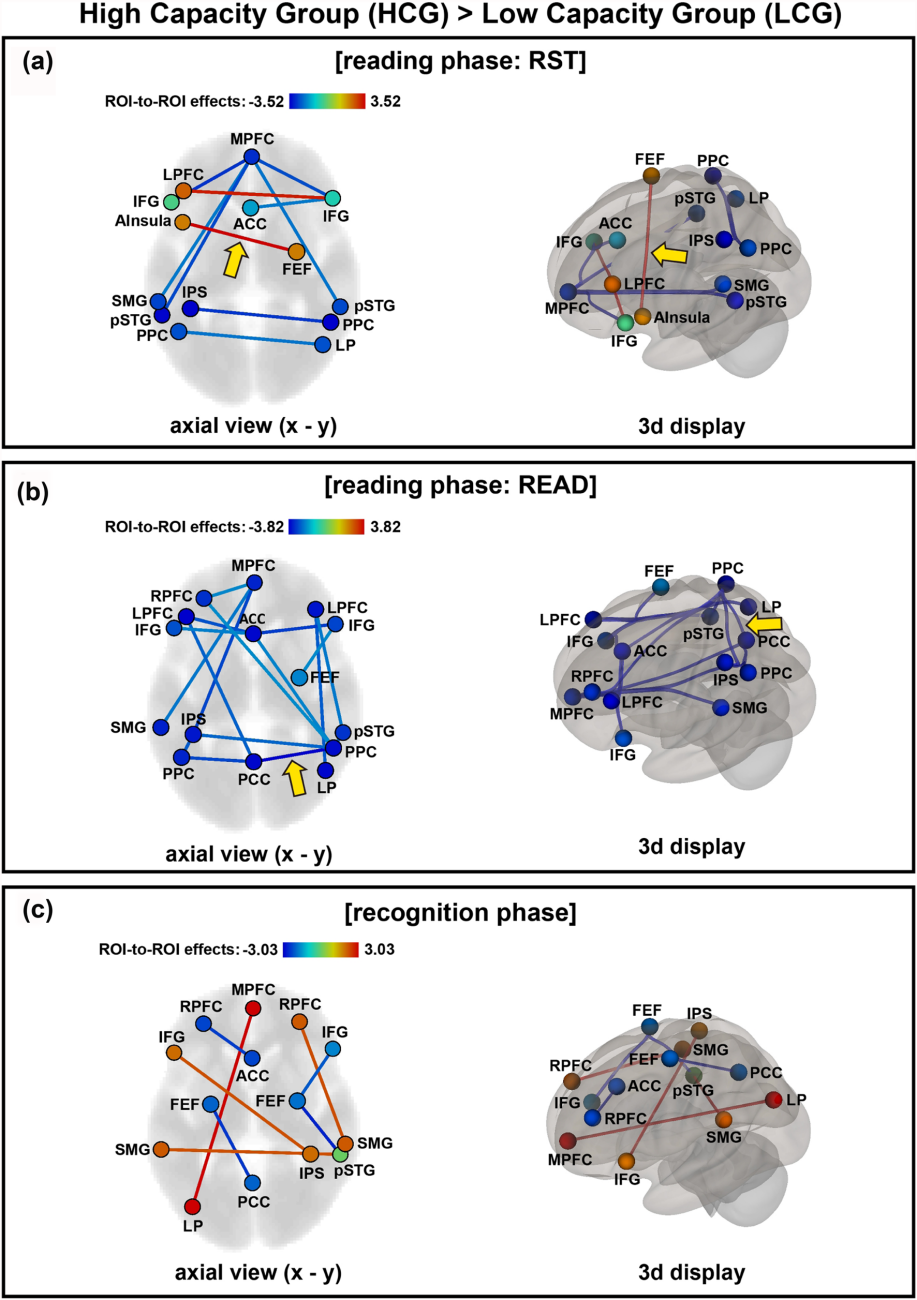
Network connectivity during the reading phase of RST condition (**a**) and READ condition (**b**), and recognition phase of RST conditions (**c**). Each figure shows network connectivity of axial (left figure) and three-dimensional brain displays (right figure). Red lines indicate higher connectivity in the high capacity group (HCG) than in the low capacity group (LCG) (uncorrected for multiple comparisons, *p* < .05). Blue lines indicate higher connectivity in the LCG than in the HCG (uncorrected for multiple comparisons, *p* < .05). In the (**a**), the yellow arrow shows network connectivity between the anterior insula (AInsura) and frontal eye field (FEF) were more strongly connected in the HCG than in the LCG (corrected, *p* < .05, p-FDR (seed-level correction)). In the (**b**), the yellow arrow shows network connectivity between the posterior cingulate cortex (PCC) and posterior parietal cortex (PPC) were more strongly connected in the LCG than in the HCG (corrected, *p* < .05, p-FDR (seed-level correction)).

During the task fMRI, network connectivity was analysed during the reading and recognition phases of the RST and reading phase of READ task. Figure 3a shows the network connectivity during the reading phase of the RST condition wherein specific and significant group differences were observed. The anterior insula (in the SN, left hemisphere) and frontal eye field (FEF; in the DAN, right hemisphere) were more strongly connected in the HCG than in the LCG (corrected, *p* < 0.05, p-FDR (seed-level correction)). Moreover, the lateral prefrontal cortex (in the FPN, left hemisphere) was more strongly connected to the left and right inferior frontal gyrus (IFG, in the Language Network: LN) in the HCG than in the LCG (uncorrected for multiple comparisons, *p* < 0.05). Additionally, Fig. 3b shows the network connectivity during the READ task. Unlike the RST condition, all regions were more strongly connected in the LCG than in the HCG during the READ task (uncorrected for multiple comparisons, *p* < 0.05). The only connectivity between PCC (in the DMN) and right PPC (in the FPN) showed corrected, *p* < 0.05 (p-FDR (seed-level correction)). Figure 3c shows the network connectivity during the recognition phase of the RST condition. The MPFC (in the DMN) and left lateral parietal (LP, in the DMN), and between right rostral prefrontal cortex (rPFC, in the SN) and right superior marginal gyrus (SMG, in the SN), and left inferior frontal gyrus (IFG, in the LN) and right inferior parietal sulcus (IPS, in the DAN) were more strongly connected in the HCG than in the LCG (uncorrected for multiple comparisons, *p* < 0.05). In addition, the left SMG (in the SN) and right SMG (in the SN) were more strongly connected in the HCG than in the LCG (uncorrected for multiple comparisons, *p* < 0.05).

**Figure 4:**
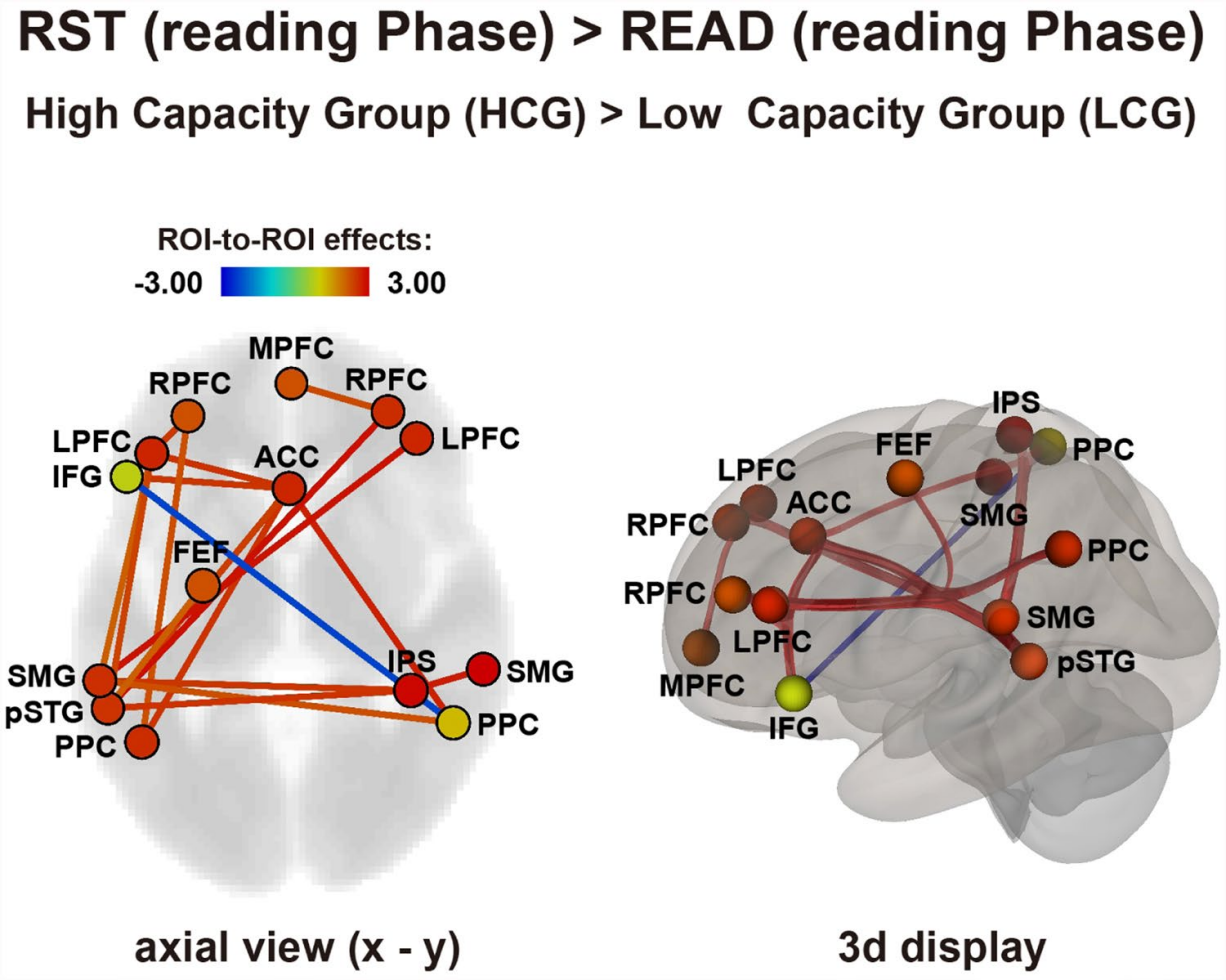
Comparison between the reading phase of the RST and READ conditions. Differences in network connectivity are shown in axial (left side figure) and three-dimensional brain displays (right side figure). Red lines indicate higher connectivity in the high capacity group (HCG) than in the low capacity group (LCG) (uncorrected, *p* < .05). Blue lines indicate higher connectivity in the LCG than in the HCG (uncorrected, *p* < .05).

We further clarified the differences in network connectivity between the RST and READ conditions by subtracting the READ condition from the RST condition (Fig. 4). Most network connectivity was stronger in the HCG than in the LCG, with the exception of the connection between the left IFG and right PCC (uncorrected for multiple comparisons, *p* < 0.05). Notably, the ACC (in the SN) and PPC in the both hemisphere (in the FPN), and between ACC and left LPFC (in the FPN) were more strongly connected in the HCG than in the LCG.

## Discussion

The present study demonstrated that both during resting and working memory task conditions, participants with high and low capacity exhibited significant differences in the connectivity of major resting-state networks. During the resting-state condition, the HCG generally showed weaker network connections between the DAN and SN than the LCG, with the exception of the connection between the MPFC and PPC. However, during the working memory task, the HCG exhibited stronger coupling between the DAN and SN, as well as between FPN and SN compared with the LCG. Thus, compared with the LCG, the HCG showed weaker connectivity between most networks during rest, but stronger connectivity during the performance of a working memory task.

These results are in agreement with those of another study that reported network connectivity variations between the DMN and DAN as the basis for differences in working memory capacity^35^. In that study, the working memory capacities of participants were estimated using a complex span test—the same span test as that used in this study, and it reported that working memory capacity was associated with a stronger MPFC-DLPFC anti-correlation. Based on a 3-back task, a significant anti-correlation between DMN and DAN was also reported to be positively correlated working memory performance^34,39^. It is also suggested that anticorrelation decrease was found in the physiological and pathological aging^37,38^.

However, in the present study, at rest, only one network connection between the MPFC and PPC was found to be stronger in the HCG than in LCG. The MPFC is a major DMN region, and the PPC is one of the FPN regions. The PPC is suggested to be involved in the shifting and focusing of attention, both of which are important for the execution of working memory tasks^40^. In addition, in our previous research, we found activation differences in PPC between high and low capacity groups during performing a difficult reading span task in which shifting attention was strongly required^18,19^. Consistent with these findings of previous studies, the PPC may play a role in shifting attention to relevant issues to support working memory task performance.

Thus, the stronger connectivity between the MPFC and PPC observed in the HCG than in the LCG suggests that individuals with a high working memory capacity are prepared to transfer attention at any time even when they are at rest.

During performance of the RST, significant between-group differences were observed in among FPN, DAN and SN connectivity, with the left PFC (in the FPN) more strongly connected to the left and right IFG (in the LN) in the HCG than in the LCG. The PFC and IFG are important to working memory performance; the PFC plays a role in executive function^11,14,15,41,42^ and the IFG is required to maintain words phonologically in working memory^43,44^. Moreover, while performing the RST, dual-task performance is needed to read sentences and maintain the target words; that is, executive control for attention shifting and focusing attention is required to manage this task. The connectivity among FPN, DAN and SN are important for dual-task performance, and a higher connectivity between these network regions leads to better performance during dual-task performance in a working memory task.

Interestingly, during the READ task (Fig. 3b), all the brain regions were more strongly connected in the LCG than in the HCG. The single task of reading required only weak connectivity for participants in the HCG; in contrast, for those in the LCG, stronger connectivity in all networks was confirmed. This result suggests that individuals with lower capacity use several networks to perform even single tasks.

Moreover, LCG was found to have higher network connections than HCG in all network connections except the DMN and PPC networks under the resting condition as well as the read condition.

One potential reason for this result is that the single task reading condition is more difficult for participants in the LCG. However, the behavioural data did not indicate a significant difference in reading time between the two groups. Moreover, the recognition accuracies of the stimulus sentences, measured after the fMRI scan, did not indicate significant differences between the two groups.

Another potential reason for this finding is that participants in the LCG use extensive network connectivity while resting or performing a simple single task, and hardly increase network connectivity during a more challenging dual task. On the contrary, the strong connectivity between the anterior insula and FEF in the RST dual-task condition was confirmed in the HCG during the RST condition. Both the anterior insula and FEF may promote task performance, as both regions play a role to attention shifting. Furthermore, the connectivity between left and right SMG, both included in the SN, was stronger in the HCG than in the LCG in the RST dual-task condition. Since the SN is involved in switching between the DAN and DMN^30,31^, these results suggest that participants in the HCG effectively switched attention using the SN, resulting in better performance accuracy compared with that in the LCG.

In addition, while performing RST, the HCG exhibited stronger coupling between the ACC and other areas related to attentional control compared with that in the READ condition (see Fig. 4). The ACC is reportedly involved in cognitive conflict monitoring during working memory performance^45–48^. A recent study also suggested that increased activity of the ACC was associated with increased functional coupling between the right middle FG and SPL while performing a working memory 2-back task^49,50^.

We previously found differences between participants with low and high capacity in terms of DLPFC and ACC activity enhancement during the RST^14,15^, as well as a stronger correlation between the DLPFC and ACC in the high score group than in the low score group^14,15^. In addition, the results of behavioural performance showed that that LCG had more intrusion errors than HCG, which is the error reporting word of the sentence other than target word, implying that participants in the LCG hardly manage conflict resolution^19^.

Following these reports, the ACC may be used as a central starting point for working memory tasks. During the implementation of a dual task, network coupling between the ACC and DAN, which is relevant to attention shifting, is required. However, this network may have been less recruited in the LCG, deteriorating their task performance. Most network connections involving the ACC were stronger in the HCG than in the LCG group. Based on these results, the ACC plays a central role as a brain hub network during the execution of dual tasks only in the HCG. On the contrary, the LCG showed increased connectivity between ACC and PFC only during the recognition phase. This increase in connectivity implies a confusion between the target word and other words in the sentences during the recognition phase, because participants in the LCG did not pay attention to the target word in the reading phase and, subsequently, faced confusion in the recognition phase. It has also been suggested that binding to ACC is reduced in patients with mild dementia^37^.

In the present study, the two groups differed on dual-task performance, but not in performance on a single-task memory span test. Thus, the connectivity differences identified in the present study are based on the executive function of working memory. However, the connectivity differences of two groups appeared both during resting and performing tasks. Some studies reported that DMN is associated with passive self-monitoring and mind wandering^51^. It is also reported that DMN activity is higher in participants who reported more mind wandering^52,53^. Future research from this perspective is desired. In addition, the number of participants in this experiment was not very large, which may be one of the reasons why no significant difference in corrected criterion was found. Future research will need to be verified by increasing the number of participants.

In conclusion, the present results demonstrate that individuals with high working memory capacity had a weaker connectivity among most brain networks at rest than those with a low memory capacity. However, even at rest, inter-regional coupling between the MPFC and PPC only appeared in the HCG, and this connectivity enables a smooth attentional shift when a cognitive task is required. During the performance of a working memory task, stronger connectivity among DAN, FPN and SN were confirmed in HCG compared with the LCG. Moreover, ACC acted as a central network hub in the HCG^54^. This stronger connectivity among DAN, FPN and SN enables participants in the HCG to have better performance in working memory task than those in the LCG.

## Methods

### Participants

A total of 38 healthy Japanese adults (26 males and 12 females), aged 20–27 years (mean ± SD = 21.6 ± 1.6 years) were recruited for the present study. All participants were recruited from the same university. All participants were right-handed. Participants met the criteria for participation in the fMRI study. The Ethical Committee of the National Institute of Information and Communications Technology and the Safety Committee of the Centre for Information and Neural Networks (CiNet) approved the experiments. All methods were performed in accordance with the relevant guidelines and regulations. All participants were paid for their participation and provided informed consent prior to participation. Four participants were excluded from data analysis due to the following reasons: one had a low score (below 50% accuracy) in the tasks and three had high sleepiness ratings (during fMRI). Thus, data from 34 participants (24 males and 10 females) were used for subsequent behavioural and fMRI analyses.

### Behavioural tests outside the scanner

To measure participants’ individual differences in working memory capacity, we conducted RST and word span testing prior to the fMRI experiment (one participant performed the tests after the fMRI).

#### RST

In this task, participants were instructed to read aloud a few sentences and simultaneously remember an underlined word (to-be-remembered target word) in the sentence. The task began with a two-sentence condition and progressed up to a five-sentence condition.

Working memory capacity was estimated using three indices: weighted value^7^, total recall scores, and span score^4,6^. The total recall score indicated the total number of target words correctly recalled (*max* = 70). The weighted value indicated the total number of target words when all target words in the trial were correctly recalled (*max* = 70). The RST span score indicated the highest level at which three out of five trials could be correctly recalled (*max* = 5.0). If the participants correctly recalled only two of five trials for a particular sentence condition, the participant was allocated a score of 0.5 for that condition.

#### Word span test

RST requires both processing and storage, whereas the WST requires only storage. In this task, participants were instructed to serially recall sequences of visually presented words. The task started with a two-word set, with each set comprising two trials. The task continued until participants failed to recall two out of two trials. The highest level at which all trials could be correctly recalled was defined as WST span score. If the participants correctly recalled only one of two trials for a particular word condition, the participant was allocated a score of 0.5 for that condition.

### Behavioural tasks in the scanner

#### Resting condition

Brain activity at rest was measured, with participants instructed to close their eyes and spend eight minutes doing and thinking as little as possible. Two sessions (Pre and Post) were performed, interspersed with a task session; however, we only analysed the resting-state brain activity in the Pre session to ensure that the resting-state data were not affected by task performance.

#### Working memory task in the fMRI scanner

Participants performed RST and READ tasks in the fMRI scanner (see Fig. 2a) using block design task procedure. In the RST condition (see Fig. 2b, left side), participants were instructed to silently read visually presented sentences, and remember an underlined word in the sentence. One block consisted of five sentences, and each sentence was presented for five seconds. Immediately after the RST blocks, in the recognition phase, participants reported the words they could remember. One set of three alternatives (two words and one “×” mark) was presented, and participants selected the appropriate alternative. If the word they remembered was present in the alternatives, they had to press the button corresponding to the position where the word was displayed. If the word they remembered was not displayed, they selected the “×” mark. Participants were instructed to respond as quickly and accurately as possible. In the READ condition (see Fig. 2b, right side), participants were instructed to simply read the sentences silently. In order to measure the time taken to read the sentences in both RST and READ conditions, participants were instructed to press a key corresponding to the arrow direction (see Fig. 2b) after reading each sentence.

Three types of blocks were performed in the task session: RST (reading phase), RST (recognition phase) and READ (reading phase). After alternately repeating the READ block (25 s) and the RST (reading + recognition) block (25 s and 12.5 s, respectively) four times in this order, an approximately 60-s interval was provided. RST (reading + recognition) and READ (reading) blocks were then alternatively repeated four times (in the opposite order before breaking). The inter-trial intervals between READ and RST were 12.5 s. The total time of the task session was approximately 14 min.

### fMRI data acquisition and analysis

Whole brain imaging data were acquired using a 3.0-T MRI scanner (MAGNETOM Trio, A Tim System 3T, Siemens Healthineers, Erlangen, Germany) with a 12-channel head coil. Head movements were minimised. For functional imaging, a gradient-echo echo planar imaging sequence with the following parameters was employed: repetition time (TR) = 2500 ms, echo time (TE) = 30 ms, flip angle = 80°, field of view (FOV) = 256 × 256 mm^2^, voxel size = 4 × 4 × 4 mm^3^, number of slices = 42, and thickness = 4 mm. There were three sessions in each imaging experiment: Resting (Pre), working memory task, and Resting (Post). The parameters of the echo planar imaging sequence were the same for all sessions. The numbers of images acquired in each session were 198, 329, and 198, respectively.

After completion of the experimental sessions, T1-weighted anatomical images were collected for anatomical co-registration using a three-dimensional magnetization-prepared rapid gradient-echo pulse sequence (TR = 1900 ms, TE = 2.48 ms, flip angle = 9°, FOV = 256 × 256 mm^2^, matrix = 256 × 256, number of slices = 208, and voxel size = 1 × 1 × 1 mm^3^).

Stimulus presentations were synchronised with the fMRI pulse at the beginning of the scanning session using Presentation software (Neurobehavioral System, San Francisco, CA). Data analysis was performed using the CONN toolbox^55^, running on SPM12 (Wellcome Department of Cognitive Neurology, London, UK) and MATLAB (MathWorks, Sherborn, MA).

All functional images were pre-processed using CONN’s default pre-processing pipeline for volume-based analyses: functional realignment and unwarping, slice-timing correction, outlier identification, direct segmentation and normalization to Montreal Neurological Institute space, and functional smoothing (8-mm)^56^. The six initial images of each scanning session were excluded from the analysis to eliminate any non-equilibrium magnetization effects.

### Connectivity analysis

After pre-processing, we implemented CONN’s default denoising pipeline to minimise the influence of residual noise components in the blood oxygenation level-dependent signal^56^. CONN uses an anatomical component-based noise correction procedure (aCompCor).

Functional connectivity was estimated by CONN’s ROI-to-ROI analyses. The connectivity between regions-of-interest (ROIs) of four main resting-state networks, namely the DMN, DAN, FPN, and SN, as well as the LN was analysed, as the participants read sentences under both RST and READ conditions. Moreover, we have measured network connections that include brain regions that have been found to be active during the performance of working memory tasks. Nineteen of CONN’s default ROIs (CONN’s ICA analyses of the Human Connectome Project dataset) were selected: “networks.DefaultMode” (four ROIs: MPFC, LP(left), LP(right) and PCC), “networks.DorsalAttention” (four ROIs: FEF(left), FEF(right), IPS(left) and IPS(right)), “networks.FrontoParietal” (four ROIs: LPFC(left), LPFC(right), PPC(left) and PPC(right)), “networks.Salience” (seven ROIs: ACC, AInsula(left), AInsula(right), RPFC(left), RPFC(right), SMG(left) and SMG(right)), and “networks.Language” (four ROIs: IFG(left), IFG(right), pSTG(left) and pSTG(right)). The ROIs involved in the “networks. DefaultMode” group comprise typical brain areas reported as important in the resting state. The ROIs involved in the “networks.DorsalAttention” group and “networks.FrontoParietal” group constituted the WMN, and the ROIs of the “networks.Language” group are considered to sustain working memory activity. The ROIs of the “networks.Salience” group are thought to be involved in the switching between the DMN and WMN.

#### Recognition test after scanning

To ensure that participants did not ignore the reading sentences and devoted themselves only to remembering the target words, a recognition test of stimulus sentences was performed, assessing whether participants recognised the sentences used in both RST and READ conditions after completion of fMRI scanning. The recognition test comprised 32 sentences: eight sentences appeared in READ blocks, eight in RST blocks, and 16 did not appear in either task block.

#### Estimation of sleepiness after the fMRI experiment

The participants were prohibited from sleeping during the resting condition. After the fMRI experiment, they rated their sleepiness during the resting sessions using four scales: (1) not drowsy at all, (2) a little drowsy, (3) about half drowsy, and (4) mostly drowsy. Three participants who rated “(4) mostly drowsy” in the Pre session were excluded from the data analysis.

## Data Availability

It is difficult to provide the data because we did not have permission to provide the data from the participants of this experiment at the time of this experiment.
